# Retraction of: The microRNA miR-29c-5p inhibits cell proliferation and migration by targeting TMEM98 in head and neck carcinoma

**DOI:** 10.18632/aging.206346

**Published:** 2025-11-30

**Authors:** Jingjia Li, Weixiong Chen, Lixia Luo, Lieqiang Liao, Xuequan Deng, Yuejian Wang

**Affiliations:** 1 Department of Otolaryngology Head and Neck Surgery, The First People’s Hospital of Foshan, Guangdong, China; 2 Department of Nosocomial Infection Control, The First People’s Hospital of Foshan, Guangdong, China

**Keywords:** microRNA, proliferation, metastasis, TMEM98, head and neck squamous cell carcinoma

**This article has been retracted: **Aging has completed its investigation of this article, following concerns that the cell migration and invasion images in Figures 3 and 6 contained duplicated panels. The authors confirmed this error but were unable to provide the original raw data necessary to verify or correct the images. As these figures directly support the article’s central conclusions regarding the biological function of miR-29c-5p and TMEM98, this issue undermines the overall validity of the study.

Furthermore, a subsequent review of the records revealed two additional serious concerns:

1. Ethical Compliance: The study, which involved human tissue samples, did not have formal approval from an Institutional Review Board (IRB) or equivalent ethics committee.

2. Informed Consent: Documentation for patient informed consent could not be verified for a subset of the cases. Given these multiple, serious concerns, all authors agree to the retraction of this article. The authors take full responsibility for these errors and deeply apologize for any negative impact on the scientific community. The Editorial decision was made to retract this article.

